# Psychometric Properties of the Existence Subscale of the Purpose in Life Questionnaire for Chinese Adolescents in Hong Kong

**DOI:** 10.1100/2012/685741

**Published:** 2012-08-13

**Authors:** Ben M. F. Law

**Affiliations:** Department of Social Work and Social Administration, The University of Hong Kong, Hong Kong

## Abstract

The current study aims to test the psychometric properties of the Existence Subscale of the Purpose in Life Questionnaire (EPIL) for early adolescence. The Purpose in Life Questionnaire (PIL), originally created by Craumbaugh and Maholick, is a 20-item scale measuring different dimensions of life purposes. The current study selected seven items representative of the existence dimension to form another scale, the EPIL. The analysis was based on 2842 early adolescents, ranging from 11 to 14 years old. Principal axis factoring found one factor, with 60% variance being explained. Cronbach's alpha for the EPIL was 0.89, which was high. The factor structure was stable across genders. Criterion-related validity was determined when the scale was used to differentiate volunteers and nonvolunteers. Construct validity was found when the scale was associated with life satisfaction. The results give support to the fact that the EPIL could be used alone to measure the psychological well-being of early adolescents and the appropriateness of the EPIL in adolescent research.

## 1. Introduction

The Prime Minister of the United Kingdom, David Cameron, attributed the August 2011 social unrest in London to one fundamental reason, the lack of morality. The drive provided by the life purpose (or life meaning) is one avenue toward human morality and meaningful existence [1]. Human existence has to tackle his or her own being and the relations among people, materials, and transcendental quests. One key factor maintaining human existence is the will to meaning. According to Frankl [2], human nature is subject to the “will to meaning.” Thus, life purposes are important to human motivation. Damon et al. [3] emphasized the distinctiveness of purpose: (a) it is a goal which is more stable and far-reaching; (b) it is a personal search but intended for a desire to make a different world or matters larger than self; (c) it is always directed toward a finished end (p. 121). Life purposes can be derived from three sources [2], namely, (a) creative work or art or scholarly endeavor, (b) deep experiences and interpersonal relationships, and (c) one's attitudes toward human suffering that cannot be avoided. Yalom [4] contended that the problem of life meaning is that a human being seems to be predestined to instill a meaning to life. However, every individual has to decide and commit one's own meaning to his or her own life. Without life purposes, an individual will experience *existential frustration*, characterized by meaninglessness, boredom, and a hollow personal existence. This is the dilemma of each individual.

The concept of purpose in life has been researched extensively. Ryff [5] has delineated that the purpose in life is a distinctive domain of psychological well-being. People with high life purposes tend to have life goals and a sense of directedness. They feel that there is a meaning to present and past lives. They hold beliefs, aims, and objectives for living. People with high life purposes also demonstrate greater self-confidence, self-acceptance, and life satisfaction [6]. Greater religiosity exists [7]. Positive mental adjustments, such as stability, maturity, responsibility, and good health, also characterize these people [8]. They cope with life stresses more effectively [9]. On the other hand, people with low life purposes tend to have suicidal ideation [10], hopelessness [11], psychopathology [12], and depression [13].

Most of the studies were conducted in the West and utilized adults as subjects [10, 14]. Few studies focused on adolescents and were implemented in non-Western contexts [15]. Worse, no studies related to purpose in life and early adolescents (ages 11 to 14 years) exist. Majority of early adolescents are studying in junior or high schools. Life purposes may be linked to academic achievement and interpersonal issues, which could be the sources of life meaning according to Frankl's formulation [2]. During this early transition period, adolescents are exposed to changes in educational, interpersonal, health, and identity issues [16]. Their life purposes may change accordingly [17]. Life purposes during adolescence can facilitate the development of prosocial behavior, moral commitment, achievement, and high self-esteem [18]. Life purposes can facilitate the formation of moral identity [19]. Thus, healthy adolescent psychosocial development should include the dimension of life purposes [20]. Based on this, an investigation of life purposes among early adolescents is needed.

One problem related to the studies of life purposes and early adolescents is the measurement issue. Generally, the most common scale used is the Purpose in Life Questionnaire (PIL) [21]. The PIL is a 20-item self-report and 7-point attitude scale which measures the extent to which respondents perceive their lives as meaningful and purposeful. Each item presents two antagonistic ends (e.g., exciting versus dull life) from which respondents have to determine their conditions. Their perception is an ontological significance of life.

The scale is relatively reliable, in terms of Cronbach's alpha (0.84) [15] and split-half reliability (0.92) [22]. One area of concern is the factor structure of PIL. Yalom [4] suggested that PIL should consist of six areas, namely, life meaning, life satisfaction, freedom, fear of death, suicide, and personal perception of life. Shek [15] obtained a five-factor solution (quality of life, goal, death, choice, and retirement) with two general factors (existence and death). Other studies have found within the PIL one general factor [23, 24] with different primary factors.

Direct use of the PIL on early adolescents may be problematic. Some items, such as the clarity of life goals, are too abstract for early adolescents. Several items, such as a reason for existence and whether the respondent has a sense of meaning in the world, may be beyond the lived experience of early adolescents. One dimension proposed by Shek [15], death, is not a topic adolescents normally think about, unless they are directly faced with it [25]. Selection of related items and formation of another scale with sound psychometric properties is more practical, especially when we want to examine the purposes in life among early adolescents.

In view of the issues arising from item relevance to early adolescents and the categories of subscales, studies related to PIL and early adolescents practically do not exist. Thus, if we want to adopt the PIL for early adolescents, the constraints cited previously should be addressed. One solution is to select relevant domains and choose the items with reference to those domains. Subscales have been used independently in personality research, such as the Big Five [26]. When part of the scale is used, the psychometric properties, such as reliability and validity of the scale, are affected.

The current study explores the psychometric properties of the Existence Subscale of the PIL (EPIL). As suggested by Shek [15], there are two general factors. Compared with the *death* factor, the *existence* factor is more relevant to adolescents and, thus, selected. Existence includes one's enthusiasm and excitement about life, a belief that one's daily activities are worthwhile, as well as a sense that one's life has meaning. One dimension of life meaning and purposes in life is the manifestation of prosocial behavior such as volunteerism [5]: people with high life purposes tend to involve more in prosocial activities. The use of volunteers and nonvolunteers can differentiate whether the instrument is sensitive to measure the existence dimension of purposes in life. Purposes in life and life satisfaction are closely connected as they share the same construct, psychological well-being [5]. The relationship between purposes in life and life satisfaction suggests construct validity.

## 2. Method

The current paper focused on the validation of the EPIL. Based on a large-scale survey in Hong Kong, the reliability, validity (criterion-related validity and construct validity), and factor structure of the scale were examined. The analyses were performed with IBM SPSS Statistics version 19.0.

### 2.1. Study Participants and Procedure

 A total of 2,842 high school students from Grades 7 to 9 in Hong Kong (ages range from 11 to 14) participated in the convenience sampling study. Among the participants, 1747 (61.5%) were girls, whereas 1095 (38.5%) were boys. The mean age of the participants was 13.33 (SD = 0.73).

Both parental and participant consents were obtained. All respondents completed the scales and demographic characteristics in a self-administration format, with adequate time provided.

## 3. Instruments

### 3.1. Existential Scale of the Purpose in Life Questionnaire (EPIL)

 Craumbaugh and Maholick [21] designed the PIL, whereas Shek [15] validated the scale in the Chinese context. For the original PIL, Craumbaugh and Maholick did not evaluate the factorial structure of the PIL. They designed items mainly to quantify the existential concept of purposes in life in relation to existential frustration. The internal factors were not their concerns. Shek's study [15] categorized items into two general concepts, namely, existence and death.

The current EPIL selected items related to the existence domain from Shek's study (PIL 1, 2, 5, 6, 8, 9, 11, 12, 16, 19). A group of adolescents in a secondary school was asked to evaluate the content of the EPIL during a pilot study. PIL 5 and PIL 19 were suggested to be removed because they thought that the meaning was very similar to PIL 2. Both statements are about the excitement of everyday life. The meaning of PIL 2 was direct and easy to grasp. Item 11 was selected because some adolescents did not get the meaning very well, that is, I often wonder why I exist. Seven items were used as the foundation of EPIL (PIL 1, 2, 6, 8, 9, 12, 16). The original numbering of the items is adopted for sake of clarity.

### 3.2. Satisfaction with Life Scale (LS)

 Diener et al. [27] designed the Satisfaction with Life Scale (LS) which was validated by Shek [28] in the Chinese context. The scale is a 5-item, 6-point Likert scale. In the current study, the reliability of the Satisfaction with Life Scale (LS) was 0.85, in terms of Cronbach's *α*.

## 4. Results

The principal axis factoring with varimax rotation resulted in a one-factor solution which explained a 60.09% variance (Table 1). The eigenvalue of the first factor was 4.21, whereas the second factor was less than the unity, that is, 0.74. The original one-factor framework could be demonstrated by this principal axis factoring. To test the stability of the factor structure, two independent principal axis factoring with varimax rotations were performed for boys and girls, respectively. One identical factor with an eigenvalue greater than the unity was obtained for both genders. The variances explained for boys and girls were 58.66% and 61.19%, respectively. Factor loadings range from  0.52 to 0.84 (Table 2). The coefficient of congruence was 0.998. The factor structure was stable across genders. 

Based on Cronbach's alpha, the reliability of EPIL was 0.89, which is very high. The reliabilities for boys' and girls' samples are 0.88 and 0.89, respectively. The Squared Multiple Correlations (SMCs) range from  0.35 to  0.62. The item-total correlations range from  0.49 to  0.74, which are in general high. The scale showed good internal consistency. Cronbach's alpha if one item is deleted ranges from  0.85 to  0.87. Table 2 shows the item statistics of the EPIL.  Table 3 shows the interitem correlation matrix; the relationships between items are significant.

Purpose in life is associated with prosocial behavior; hence, criterion-related validity was determined along this line of thinking. Two groups of respondents were identified. The first group included those who have volunteered in the past 12 months (volunteers), and the second comprised those who have not volunteered in the past 12 months (nonvolunteers). The mean of the EPIL for volunteers was 4.99 (SD = 1.14), whereas that for nonvolunteers was 4.75 (SD = 1.28). The univariate analysis showed that the mean EPIL of volunteers is significantly higher than that of the nonvolunteers (*t* = 5.14, *P* < 0.001). The effect size, Cohen's d, was 0.12, which is a small value. The criterion-related validity was attained.

Psychological well-being is associated with life satisfaction [5]. The EPIL is a component of psychological well-being; hence, such association with LS is hypothesized. Construct validity was performed along this dimension. The result showed a correlation between EPIL and LS of 0.56. This correlation is moderately high. All individual items are associated with LS (Table 3). The construct validity was attained.

## 5. Discussion

 The current study selected seven conceptually linked items from Shek's original Chinese version of the PIL [15] to form the EPIL. The psychometric properties were explored. The EPIL attained high internal consistency (0.89) and high item-total correlation (0.53). The reliability of the EPIL was very high, compared with those of recent studies [14]. The principal axial factoring showed that one factor could be extracted from the scale. The coefficients of congruence analysis showed that the factor can be replicated across boys and girls. The variance explained was greater than 60%, considerably higher than that of a similar study conducted with adolescents aged 11 to 20 with the 20-item full scale [15]. Thus, the EPIL is powerful in explaining the variance. The factor structure was stable across genders. The criterion-related validity was derived when the EPIL scores between volunteers and nonvolunteers were compared. The construct validity was attained when the EPIL score was highly associated with the LS score.

 The major difference between EPIL and PIL is that EPIL is a subset of the PIL. The EPIL consists of seven items from the existence domain of PIL. The psychometric properties are empirically validated in this study.

The existence dimension refers to an individual perception of his or her life. Life has meaning under all circumstances. Frankl contended that our main motivation for living is the will to find a life meaning. We have the freedom to find meaning in what we do and in what we experience. The existence dimension of the purpose in life includes whether life is perceived to be enthusiastic versus boring, exciting versus monotonous, or new versus unchanged. Youth researchers can use the scale to examine the concept of life meaning among early adolescents. A norm table can be designed to examine the trend of the purpose in life among early adolescents as well as for cross-cultural comparison. The EPIL provides an avenue for unique and down-to-earth application of measuring life purposes. The *existence* domain of PIL is more relevant to adolescents. Other domains such as death and retirement are not entirely relevant to early adolescents [15, 24]. The EPIL provides a practical approach to measure early adolescents' purposes in life.

The current study has several limitations. First, the research findings are based on the perceptions of early adolescents in Hong Kong. There is a need to replicate the current study in adolescents with different ethnicities and contexts. Second, the respondents are from convenience sampling and not from sampling, although the sample size is large. The application of the findings to other adolescent populations should be interpreted with caution because of questionable generalizability. Third, items specifically related to early adolescents' purposes in life are not included. One example is the inclusion of the importance of the academic achievement, which is demonstrated to be one of the utmost concerns among adolescents [29]. Fourth, most existing studies adopt the PIL, which differs from the EPIL. Thus, EPIL scores cannot be compared with past PIL scores directly. Despite these limitations, the current study is the first to validate the EPIL for early adolescents. The measure can be used as outcome indicators in positive youth development programs in Chinese contexts. In fact, in the Project P.A.T.H.S., measures derived from the PIL were used to assess the existential well-being of Chinese adolescents in Hong Kong [30–34].

## Figures and Tables

**Table 1:** Total variance explained and eigenvalues.

Factor	Eigenvalues	Variance explained
1	4.21	60.09%
2	0.74	10.54%
3	0.61	8.71%
4	0.43	6.16%
5	0.42	5.92%
6	0.32	4.50%
7	0.29	4.08%

**Table 2:** Items statistics of existence subscale of purpose in life scale (*n* = 2, 842).

Items equivalent to PIL	mean	SD	median	mode	SE	Corrected item-total correlation	Squared multiple correlation	Cronbach's alpha if item deleted	Factor loading
(1) I am usually completed	5.02	1.38	5.00	5	0.03	0.74	0.60	0.85	0.80
Bored—enthusiastic									
(2) Life to me seems always	4.97	1.41	5.00	5	0.03	0.70	0.58	0.86	0.77
Exciting—completely routine									
(6) If I could choose, I would	5.14	1.74	5.00	7	0.03	0.71	0.51	0.85	0.76
prefer never to have been—									
embrace my current life									
(8) I achieving life goals, I made	4.47	1.29	5.00	5	0.02	0.59	0.40	0.87	0.64
no progress—progressed to									
complete fulfillment									
(9) My life is empty, filled with	4.99	1.54	5.00	5	0.03	0.78	0.62	0.85	0.84
despair, running over with good									
things									
(12) As I view the world in	4.62	1.55	5.00	5	0.03	0.71	0.52	0.85	0.77
relation to my life, the world									
completely confuses me—fits									
meaningfully with my life									
(16) With regard to suicide, I have	5.15	1.97	6.00	7	0.04	0.49	0.35	0.88	0.52
thought of it seriously as a way									
out—never given it a second									
thought									

**Table 3:** Interitem correlation matrix.

	PIL 1	PIL 2	PIL 6	PIL 8	PIL 9	PIL 12	PIL 16
PIL 2	.71***						
PIL 6	.59***	.58***					
PIL 8	.48***	.46***	.48***				
PIL 9	.66***	.64***	.63***	.57***			
PIL 12	.58***	.56***	.58***	.53***	.65***		
PIL 16	.40***	.35***	.45***	.32**	.44***	.42***	
LS	.47***	.45***	.42***	.45***	.50***	.46***	.31**

PIL: purpose in life items; LS: satisfaction with life scale.

***P < .001.

**P < .01.
